# Modeling and Targeting *MYC* Genes in Childhood Brain Tumors

**DOI:** 10.3390/genes8040107

**Published:** 2017-03-23

**Authors:** Sonja Hutter, Sara Bolin, Holger Weishaupt, Fredrik J. Swartling

**Affiliations:** Department of Immunology, Genetics and Pathology, Science for Life Laboratory, Rudbeck Laboratory, Uppsala University, 751 85 Uppsala, Sweden; sonja.hutter@igp.uu.se (S.H.); sara.bolin@igp.uu.se (S.B.); holger.weishaupt@igp.uu.se (H.W.)

**Keywords:** MYC, oncogene, pediatric brain tumors, targeted therapy, medulloblastoma, glioma

## Abstract

Brain tumors are the second most common group of childhood cancers, accounting for about 20%–25% of all pediatric tumors. Deregulated expression of the MYC family of transcription factors, particularly *c*-*MYC* and *MYCN* genes, has been found in many of these neoplasms, and their expression levels are often correlated with poor prognosis. Elevated c-MYC/MYCN initiates and drives tumorigenesis in many *in vivo* model systems of pediatric brain tumors. Therefore, inhibition of their oncogenic function is an attractive therapeutic target. In this review, we explore the roles of MYC oncoproteins and their molecular targets during the formation, maintenance, and recurrence of childhood brain tumors. We also briefly summarize recent progress in the development of therapeutic approaches for pharmacological inhibition of MYC activity in these tumors.

## 1. Introduction

Brain tumors are the leading cause of cancer-related deaths among children. Primary brain tumors comprise a diverse group of neoplasms arising from different cells of the central nervous system (CNS) that can be separated according to their glial or non-glial origins. MYC family proteins (c-MYC, MYCN, and MYCL) are misregulated in various malignant brain tumors in children and adults [1,2].

The MYC family proteins are basic helix-loop-helix leucine zipper transcription factors with a crucial role in proliferation, differentiation, cell cycle progression, metabolism, and cell survival/apoptosis [3,4]. The MYC transcription factors form heterodimers with their partner protein MAX and bind to DNA at Enhancer box (E-box) sequences (the canonical CACGTG and other non-canonical sites) to activate the transcription of target genes. By associating with a second transcription factor, MIZ-1, MYC can also function as a transcriptional repressor [5].

In normal cells, MYC expression is tightly regulated (at the transcriptional and post-transcriptional level) by developmental and mitogenic signals. MYC oncogene deregulation is observed in more than half of human cancers as a consequence of gene amplification, overexpression, chromosomal translocation, and/or protein stabilization [4,6]. The ensuing high MYC levels are not only able to drive tumor initiation, progression, and recurrence, but are also necessary for tumor maintenance. Targeted MYC inactivation in tumors that are dependent on *MYC* genes often leads to growth arrest, apoptosis, and differentiation [7].

This review focuses on the role of MYC proteins and their regulatory network in malignant brain tumors. We will also describe ways of modeling MYC-driven brain tumors and highlight recent findings describing the attempts to target MYC proteins.

## 2. Pediatric Brain Tumors and Clinical Features

**Figure 1:**
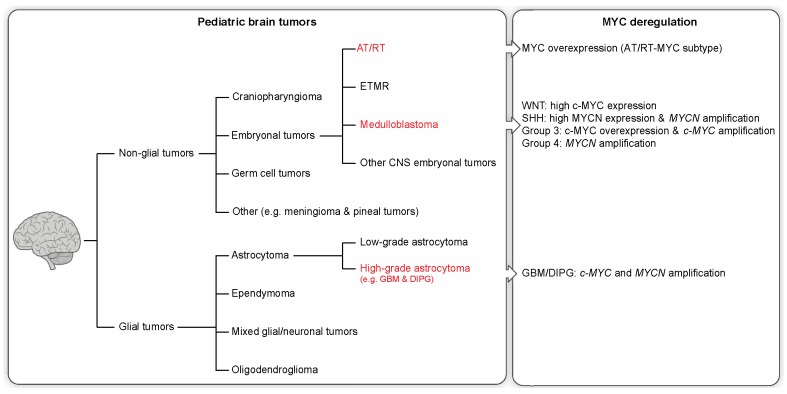
The most common brain tumors in pediatric patients. Brain tumors are a heterogeneous group of neoplasms divided into two broad groups, glial and non-glial tumors. Entities with known MYC dysregulation are highlighted in red. AT/RT: atypical teratoid/rhabdoid tumor. ETMR: embryonal tumor with multilayered rosettes. GBM: glioblastoma. DIPG: diffuse intrinsic pontine glioma.

Cancer is the second most common cause of death in children, surpassed only by accidents. In children, CNS neoplasms are the most common solid tumor type and the second most common childhood malignancy after leukemia [8]. In 2014, brain cancer surpassed leukemia to become the leading cause of cancer-related deaths in children as a result of improved leukemia treatment [9]. Primary brain tumors can be categorized as either glial or non-glial tumors (see Figure 1).

### 2.1. Non-Glial Tumors

Non-glial brain tumors include embryonal tumors, craniopharyngioma, germ cell tumors, and other rare entities. Embryonal tumors are the most common malignant CNS neoplasms in children (~15%) [10] and are composed of undifferentiated (small round) or poorly differentiated cells similar to the ones in the developing embryo. Tumors within this group include medulloblastoma, atypical teratoid/rhaboid tumors (AT/RT), ETMR (embryonal tumor with multilayered rosettes), and other CNS embryonal tumors (previously known as CNS primitive neuroectodermal tumors (PNETs)). Despite sharing a common histological pattern, embryonal tumors are biologically distinct.

Medulloblastoma is by far the most common form of embryonal tumors in children (ages 0–14 years), accounting for 63% of all embryonal CNS neoplasms [10]. These tumors commonly originate in the cerebellum or posterior fossa and tend to disseminate via the cerebrospinal fluid (CSF). Amplification and overexpression of the MYC oncogene family, especially c-MYC and/or MYCN, have been described in medulloblastoma. Patients whose tumors exhibit *MYC* gene family amplification usually have a significantly worse prognosis [11].

CNS AT/RT are rare, but highly malignant embryonal tumors in infants [12]. AT/RTs represent only 1%–2% of all pediatric CNS tumors, but account for up to 10%–20% of brain tumors in children younger than three years of age. These tumors occur in both supratentorial and infratentorial brain regions, but are predominantly observed in the supratentorial region.

Embryonal tumor with multilayered rosettes (ETMR) is a recently described entity of embryonal tumors that encompass embryonal tumor with abundant neurophil and true rosettes (ETANTR), medulloepithelioma, and ependymoblastoma. Despite presenting as distinct histological variants, these tumors share a characteristic molecular signature (amplification of a large microRNA cluster on chromosome 19 known as C19MC) and are thus considered a single entity [13]. ETMRs arise predominantly in children under four years of age and are associated with a dismal prognosis.

Another tumor type derived of non-glial origin is craniopharyngioma, which accounts for 4% of all brain tumors in children [10]. These are benign (World Health Organization (WHO) grade I), slow-growing, partially cystic epithelial tumors found in the sellar or suprasellar region surrounding the pituitary gland in the brain.

Intracranial germ cell tumors are a heterogeneous group of rare neoplasms that constitute about 3% of childhood brain tumors in the USA and Europe, but in Japan and other Asian countries an incidence of up to 11% of pediatric CNS tumors has been reported [14]. These brain tumors are most commonly found in the pineal and suprasellar region in the brain [14].

### 2.2. Glial Tumors

Glial tumors make up approximately 53% of all pediatric brain tumors [10] and include astrocytoma, oligodendroglioma, glioblastoma, ependymoma, and a few rare histologies. Most of the glial tumors in children are slow-growing pilocytic astrocytomas or other low-grade tumors (WHO grade I and II), accounting for over 30% of CNS tumors in this age group. High-grade gliomas (HGGs), in particular glioblastomas (GBMs), diffuse pontine gliomas, and other malignant astrocytomas account for ~19% of pediatric brain tumors (≥14 years of age) [10]. Despite occurring less frequently than their adult counterparts, pediatric HGGs nonetheless contribute substantially to childhood cancer mortality. In addition, pediatric HGGs are clinically and biologically distinct from adult gliomas [15]. GBM, which is the most common and most malignant (WHO grade IV) brain tumor in adults, comprises only 3% of all brain and CNS tumors among children aged 0–14 years [10]. Patients diagnosed with GBM face a dismal prognosis, with a median survival of only 12–15 months [16]. All HGGs share common features of high mitotic activity, marked vascular endothelial proliferation, and focal necrosis.

Diffuse intrinsic pontine gliomas (DIPGs) are highly aggressive tumors that start growing in the brainstem (pons) and infiltrate adjacent healthy tissue. DIPG account for 80% of pediatric brainstem tumors and primarily affect young children with a median survival of less than one year [17,18].

Anaplastic astrocytoma (AA; WHO grade III) is a rare, malignant, diffusively infiltrating neoplasm with nuclear atypia and increased mitotic activity, but lack vascular proliferation and necrosis, unlike glioblastomas (WHO grade IV). These tumors occur rarely in pediatric patients, accounting for only 1.5% of childhood CNS tumors [10].

Ependymomas are glial tumors that arise from ependymal cells that line the ventricles of the brain and the central canal of the spinal cord. In children, ependymal tumors account for 5.7% of primary CNS neoplasms [10] with two-thirds arising in the infratentorial region and one-third within the supratentorial compartment.

Mixed neuronal-glial tumors, which are characterized by proliferation of neoplastic cells showing a mixture of neuronal and glial differentiation, and oligodendroglioma, which are derived from oligodendrocytes, are glial brain tumors accounting for 5.7% and 0.8% of CNS neoplasm, respectively [10].

## 3. Molecular Profiling and MYC Misregulation in Childhood Brain Tumors

In recent years, comprehensive molecular profiling studies have identified distinct biologically and clinically relevant subgroups of different brain tumors entities. This section will focus on the molecular profiling of various childhood brain tumors associated with MYC dysregulation.

Medulloblastoma was formerly thought of as a single histological entity, but is now known to compromise four different subgroups with distinct biological and clinical features: Wingless (WNT), Sonic Hedgehog (SHH), and the less well molecularly characterized Group 3 and Group 4 [11,19,20]. These molecular subgroups relate to differences in age and gender distribution, rates of metastatic dissemination, and somatic alterations [20,21]. Further classification of a medulloblastoma into any of these four subgroups will provide an improved prediction of clinical outcome. The *MYC* genes are often overexpressed or amplified in medulloblastoma, with differential expression of *c-MYC* and *MYCN* among the four subgroups [1]. c-MYC is highly expressed in WNT tumors, which do not have *c-MYC* gene amplification, whereas Group 3 medulloblastomas are often associated with *c-MYC* amplification (~16%–17%) and the worst overall prognosis [1,22]. High-level expression and amplification (~8%–9%) of *MYCN* occur in SHH medulloblastoma, with amplification being predictive of a worse prognosis [1,11,22]. Moreover, some Group 4 tumors are also associated with *MYCN* amplification (~6%–7%) despite generally exhibiting low *c-MYC* and *MYCN* expression levels [1,22].

The genetic hallmark of AT/RTs is the deletion and/or mutation of the tumor suppressor gene *SMARCB1* (*INI1/hSNF5*) present in the vast majority of these tumors, resulting in a loss of nuclear SMARCB1/INI1 protein expression. Apart from these recurrent *SMARCB1/INI1* alterations, AT/RTs have a remarkably low mutation rate [23]. The rare cases of AT/RT tumors exhibiting retained SMARCB1/INI1 expression have been associated with mutations and allelic loss of the *SMARCA4/BRG1* gene [24]. SMARCB1/INI1 and SMARCA4/BRG1 are essential components of the ATP-dependent SWI/SNF chromatin-remodeling complex involved in the transcriptional regulation of a variety of genes that control cellular proliferation or differentiation [25]. Recently, a study has identified three epigenetically/molecular distinct subgroups of AT/RTs (tyrosinase (TYR), SHH, and MYC) [2]. AT/RT-TYR tumors usually occur in patients younger than one year of age with preferential infratentorial location. This subtype is characterized by large/broad *SMARCB1/INI1* deletions and overexpression of melanosomal genes, such as *TYR*, *MITF*, or *DCT* [2]. In the AT/RT-SHH subgroup, tumors are characterized by focal *SMARCB1/INI1* aberrations and overexpression of SHH pathway genes, including MYCN and GLI2 overexpression [2]. These neoplasms occur in both supratentorial and infratentorial locations. The AT/RT-MYC subtype is characterized by overexpression of the *MYC* oncogene as well as *HOX* cluster genes and focal *SMARCB1/INI1* deletions [2]. Clinically, these tumors present in older children and occur mainly in supratentorial locations.

Other CNS embryonal tumors (previously known as CNS-PNETs) account for only 2%–3% of all childhood brain tumors [26]. These tumors show an aggressive clinical behavior and a poor outcome with 50%–60% overall survival [27]. Sturm et al. recently performed an integrated genomic analysis of 323 CNS-PNETs cases and were able to cluster these tumors into non CNS-PNET tumors [28]. For example, 9% of the CNS-PNETs where clustered to HGG-MYCN tumors [28]. Moreover, the CNS-PNET cases further clustered to other types of HGGs, ETMRs, AT/RTs, and medulloblastoma as well. Interestingly, when sorting these tumors out a number of new molecular entities emerged, which could be identified as “CNS neuroblastoma with *FOXR2* activation” (CNS NB-*FOXR2*), “CNS Ewing sarcoma family tumor with *CIC* alteration” (CNS EFT-*CIC*), “CNS high-grade neuroepithelial tumor with *MN1* alteration” (CNS HGNET-*MN1*), and “CNS high-grade neuroepithelial tumor with *BCOR* alteration” (CNS HGNET-*BCOR*) tumors [28].

The major insights into GBM molecular subtypes originate from classifications studies on adult patients, which suggest two major disease entities reflecting *IDH1* mutation status [26,29,30] and a total of 3–7 molecular subtypes based on expression or methylation data [29,30,31,32,33,34]. However, pediatric GBMs have been shown to present with distinct histopathological features [35,36,37], and less is known about their molecular subtypes. Nevertheless, recent studies suggest a substantial overlap with the most robust adult expression subtypes, termed proneural and mesenchymal [15,34,37], as well as with signature mutation derived subtypes defined by the status of *IDH1* or the histone H3.3 variant encoding *H3F3A* gene [33,37], the latter of which presents a frequent phenotype reported to account for 30%–50% of pediatric GBMs [33,38]. Interestingly, a subset of proneural GBMs have previously been demonstrated to be enriched for *MYC* amplifications [31], while mutations in histone H3.3 have been suggested to be driving tumorigenesis via stimulation of *MYCN* overexpression, especially in pediatric GBMs [16]. In addition, one of the marked differences between pediatric and adult mutational landscapes is an increased focal amplification rate of both c-*MYC* and *MYCN* [15,37]. Together with the observation that the expression of a stabilized variant of *MYCN* in forebrain-derived NSCs can induce malignant glioma in mice [39], these observations suggest a role for *MYC* genes in tumorigenesis of pediatric GBM.

The genomic landscape of DIPGs has previously been shown to harbor focal amplifications of both *c-MYC* and *MYCN* [40]. Recent efforts have clustered these tumors into three predominant molecular subgroups referred to as “MYCN”, “Silent”, and “H3-K27M” [17], the last of which can be further dissected into a subset of cases harboring a K27M mutation in histone H3.3 or histone H3.1, respectively [17,41]. While the MYCN group was characterized by an amplification and accompanying overexpression of *MYCN*, the H3-K27M also exhibited an exclusive enrichment of focal amplifications of *PVT-1/MYC* [17,42], suggesting differential roles of MYCN and c-MYC in DIPG tumorigenesis.

Due to their rarity, less is known about molecular subtypes of anaplastic astrocytoma. Instead, these tumors are often classified in combination with low-grade gliomas [43], or in the general context of anaplastic gliomas [44] or HGGs [32,35]. Currently, anaplastic astrocytomas are separated into subsets based on mutations in *IDH1*, *ATRX* and *TP53*, as well as codeletion of chromosomes 1p and 19q [45]; however, the role of MYC in these subtypes is largely unexplored. Screens including either grade II and grade III gliomas [43] or pediatric HGGs [35,36,37,46] have demonstrated amplifications of both c-*MYC* as well as *MYCN*, and in the context of pediatric HGG a clear association of *MYCN* amplifications with anaplastic astrocytomas was reported [46]. Additionally, a recent mouse model has demonstrated the potential of MYC to induce gliomagenesis in mature astrocytes [47], while a comparison of paired grade II and grade III gliomas has suggested a role of MYC in driving glioma progression [48]. Further, *Trp53* mutations contribute to gliomagenesis by allowing the overexpression of *c-Myc* through downregulation of its post-translational regulator, the ubiquitin ligase Fbxw7. Expression of *Trp53* mutants or knockdown of Fbxw7 in *Pten^−/−^*;*Cdkn2a^−/−^* neural stem cells resulted in re-expression of *c-Myc* with enhanced tumorigenicity [49].

## 4. Mechanisms Involved in MYC-Driven Brain Tumor Initiation

*MYC* is one of the key oncogenes implicated in the pathogenesis of human tumors. However, surprisingly, *MYC* activation alone is not sufficient to induce tumorigenesis. Instead, MYC overexpression in normal cells can have destructive outcomes, such as proliferative arrest [50], apoptosis [51,52,53], and cellular senescence [54]. In the early 1980s, an in vitro study of *c-MYC* revealed its potential to transform primary rat embryonic fibroblasts only in cooperation with other oncogenes (e.g., *RAS*) [55]. In the following 20 years, several studies confirmed that deregulated *c-MYC/MYCN* expression collaborates with other genetic alterations to circumvent multiple intrinsic tumor-suppressing mechanisms (which inherently prevent *MYC* activation from initiating tumorigenesis) in order to start forming tumors in vivo.

Tumorigenesis requires the ability to disable the MYC-mediated apoptotic program. MYC overexpression leads to p53-dependent or p53-independent apoptosis. MYC has been shown to rapidly induce p14^ARF^ [56] which, in turn, effectively stabilizes p53 thereby activating the p53 pathway by binding to and inhibiting the MDM2 oncogene. A loss of either of these tumor suppressors accelerates tumorigenesis in MYC-driven mouse models (for more details see the next section) [57,58]. For example, human MYC-driven medulloblastoma often exhibit monoallelic loss of *TP53* typically as the result of the formation of an isochromosome 17q [19,59]. Furthermore, MYC also promotes apoptosis by disrupting the balance of pro-apoptotic and anti-apoptotic factors. High levels of MYC suppress the expression of the anti-apoptotic BCL-2 and BCL-XL [60], while at the same time inducing pro-apoptotic proteins like BIM and BAX [61,62]. For example, enforced BCL-2 oncogene expression inhibits MYC-induced apoptosis and cooperates with MYC to induce neoplastic transformation in murine lymphomas and medulloblastomas [60,63].

In the absence of a functional apoptotic response, the strong proliferative signal caused by MYC deregulation can further trigger tumor formation. MYC mRNA and protein expression levels strongly correlate with the cell proliferation rate: The MYC protein is a so-called “immediate early” gene product that can be rapidly induced in response growth factor stimulation. Ectopic c-MYC overexpression can prompt quiescent cells to re-enter the cell cycle independent of any growth stimulus [64]. MYC can stimulate cell cycle progression through several mechanisms. Firstly, MYC induces the expression of several cyclins, cyclin-dependent kinases (CDKs), and E2F transcription factors, which are involved in S phase entry. These factors are essential cell cycle regulatory proteins whose deregulation often occurs during the development of tumors. For example, cyclin D2 (CCND2) expression has been shown to be directly regulated by MYC [65]. Secondly, MYC counteracts the activity of p21^CIP1^ and p27^KIP1^ CDK inhibitors by different mechanisms, thus overcoming actions from the growth-inhibitory signal TGF-β. Lastly, MYC induces transcription of factors related to DNA replication and directly interacts with the pre-replicative complex, controlling the initiation of DNA replication and origin activity [66].

Furthermore, MYC over-activation can induce genomic instability, a process linked to tumor initiation. In vivo and in vitro models have shown that c-MYC overexpression can initiate karyotypic (chromosomal) instability [67,68,69] or locus-specific genomic instability [69,70]. The latter was first described for the *dihydrofolate reductase (DHFR)* gene, which was amplified as a result of inducible c-MYC overexpression [70]. As will be discussed in a later section, upon MYC inactivation in conditional mice models most tumors undergo proliferative arrest, differentiation, and apoptosis [7]. However, some tumors can become independent of MYC overexpression by acquiring additional genetic events such as chromosomal aberrations [67,68,71].

MYC is also linked to all key aspects of metabolic reprogramming in tumorigenesis, including the induction of glycolysis, enhanced glutamine metabolism, and lipogenesis, as well as mitochondrial biogenesis [72,73]. In addition, MYC directly regulates ribosomal biogenesis and function and consequently causes enhanced protein synthesis [72,73]. Together, these MYC-induced metabolic alterations lead to rapid cell mass expansion and hence to tumor growth. The importance of enhanced ribosome function and protein synthesis during MYC-induced tumorigenesis has been demonstrated by a study showing that diminished expression of the ribosomal protein RPL24 in transgenic mice markedly decreases MYC-induced lymphomagenesis [74].

Finally, MYC influences the tumor microenvironment, including the activation of angiogenesis and suppression of the host immune response. MYC induces the expression of the proangiogenic factors vascular endothelial growth factor (VEGF) and angiopoietin*-*2 (ANG-2) and downregulates anti-angiogenic factors like thrombospondin-1 (TSP-1) [75,76,77]. For example, in a MYC mouse model of pancreatic cancer, sustained activation of c-Myc in β cells triggers increases the expression of the inflammatory cytokine interleukin-1β (IL-1β). Secretion of IL-1β leads to the release of VEGF sequestered in the extracellular matrix, presumably via the activation of matrix metalloproteases. VEGF-A localizes to its cognate receptor on the adjacent endothelial cells, promoting their proliferation [78].

As we will discuss in more detail later, MYC inactivation results in tumor regression in most mouse models. It has been shown that complete tumor clearance following the inactivation of MYC oncogene requires the secretion of thrombospondin-1 [79] and the recruitment of CD4^+^ T cells [80]. Recent evidence suggests that MYC regulates the expression of CD47 and PD-L1, two important immune checkpoint proteins on the tumor cell surface [81]. By downregulating these checkpoint proteins MYC inhibition enhanced the antitumor immune response, but upon enforced expression of CD47 or PD-L1 tumors continued to grow.

## 5. MYC-Driven Models of Brain Tumors

Genetically engineered mouse models (GEMMs) as well as Patient-Derived Xenograft (PDX) models are indispensable tools for studying human brain tumors. These animal models are important to validate the genetic events and molecular mechanisms that contribute to oncogenesis within the CNS and to evaluate potential therapeutic strategies.

As discussed in the previous section, MYC overexpression alone is not sufficient to induce tumorigenesis in vivo. Using the RCAS-TVA system to model medulloblastoma, Fults et al. showed that c-MYC overexpression in Nestin-expressing cells was insufficient to induce medulloblastoma formation, but that it generated nests of undifferentiated cells instead [82]. However, ectopic expression of either human c-MYC or MYCN (mutationally stabilized and wild-type) in conjunction with SHH expression in Nestin-expression progenitor cells generated SHH medulloblastoma at a significantly increased incidence compared with infection with RCAS-SHH alone [83,84]. Furthermore, somatic gene transfer of c-MYC and antiapoptoic Bcl-2 induces large cell/anaplastic (LCA) medulloblastoma in Nestin-TVA mice [63]. Ectopic expression of c-MYC could also, in collaboration with RE1-silencing transcription factor/neuron-restrictive silencer factor (REST/NRSF), block the neuronal differentiation of granule progenitor cells and drive medulloblastoma in mice [85]. In addition, enforced expression of *N-Myc* in cerebellar granule neuron precursors (CGNPs) collaborates with the loss of *Trp53* and *Cdkn2c* (p18^Ink4c^) [86], as well as the loss of *Ptch1* [87] to accelerate SHH medulloblastoma development in mice.

Additionally, forced expression of wild-type *MYCN* can promote SHH-independent medulloblastoma development when driven from the brain-specific Glutamate transporter 1 (*Glt1*) promoter in the Tet-inducible Glt1-tTA;TRE-MYCN/Luciferase (GTML) transgenic model [88]. The GTML model gives rise to tumors that mostly (>80%) resemble Group 3 medulloblastoma but also smaller sets of WNT, SHH, and Group 4 tumors accentuating MYCN as a pleiotropic transcription factor in medulloblastoma tumorigenesis [88,89,90]. Most GTML tumors displayed classic or LCA histology and were Atoh1/Math1-negative. Atoh1/Math1 is a bHLH transcription factor required for SHH medulloblastoma development. Later, by using the RCAS-TVA system, we showed that specific Atoh1-negative/glial fibrillary acidic protein (GFAP)-positive brain stem cells could generate MYCN-driven medulloblastoma by using a mutationally stabilized MYCN^T58A^ viral construct. Interestingly, MYCN drives either SHH-dependent or SHH-independent medulloblastoma as a consequence of the timing of its oncogenic expression from embryonic or postnatal cerebellar stem cells, respectively [39]. It was also evident that MYCN could promote the formation of glioma from GFAP-positive stem cells isolated from the forebrain ventricular zone (VZ). The glioma formation is in line with previous results showing how transgenic c-MYC expression from GFAP positive forebrain cells give rise to gliomas [91] and further argue for a window of vulnerability during astrocyte development where c-MYC or MYCN overexpression is sufficient to trigger this neoplastic process.

In 2012, two groups described other mouse models that recapitulate many features of human Group 3 medulloblastoma. Pei et al. introduced a mutationally stabilized *c-Myc* (c-Myc^T58A^) and dominant negative *Trp53* (*DNp53*) in postnatal stem cells expressing Prominin-1 (CD133) but lacking expression of lineage-specific markers defining CGNPs [57].Using *c-Myc*^WT^ constructs also induced tumor formation in conjunction with DNp53, but with reduced penetrance and increased latency. Immunocompromised mice injected with transduced cells developed (largely undifferentiated) tumors that resembled human LCA medulloblastoma histology and gene expression profiles, different from SHH-based mouse models. MYC withdrawal caused complete tumor regression, proposing a strong *MYC* oncogene addiction in this tumor model. In contrast, Kawauchi et al. retrovirally introduced c-Myc into *Trp53^−/−^;Cdkn2c^−/−^* GNPs prior to orthotopic injection into cerebral cortices of naive recipient mice [58]. The Myc-induced tumors were distinct from WNT and SHH models. Although the neuronal lineage marker Atoh1 was used to sort for CGNPs, the resulting tumor cells had lost Atoh1 expression and instead displayed increased expression of Prominin1 and other stem cell factors. The Group 3 medulloblastoma mouse models from both these studies demonstrate that the *MYC* oncogene has to be activated either in stem cells or that the progenitor cell has to be reprogrammed by MYC for transformation to occur.

Remarkably, enforced expression of MycN in GNPs from *Trp53^−/−^;Cdkn2c^−/−^* mice induce SHH medulloblastoma, whereas expression of Myc in the same GNPs induces Group 3 medulloblastoma [58]. Recently, a subsequent study using different genetically engineered mice showed that the MYC’s binding partner MIZ-1 protein plays a pivotal role in determining the tumor identity [92]. C-MYC and MYCN are distinguished by their ability to bind MIZ-1, which shows a strong binding affinity for c-MYC but not MYCN [92]. The strong c-Myc/Miz-1 complex drives Group 3 tumors by repressing expression of genes involved in neuronal differentiation, ciliogenesis (genes responsible for assembling primary cilia) and the TGF-β pathway, thereby maintaining a stem cell-like gene expression profile [92]. In experiments employing a *c-Myc* mutant (Myc^V384D^) specifically impaired in Miz-1 binding, fewer mice developed brain tumors and tumor formation took longer [92]. In contrast, the weaker MycN/Miz-1 complex plays a role in SHH tumor development since inhibition of MycN/Miz1 binding blocked development of SHH medulloblastoma [92]. These data suggest that targeting the Myc–Miz1 complex may provide an alternative strategy for targeting MYC-dependent (Group 3) tumors.

Other Group 3 animal models were generated by overexpression of *Gfi* or *Gfi1B* and *c-Myc* in neural stem cells. Using whole genome sequencing (WGS) of primary medulloblastoma, Northcott et al*.* had identified a series of diverse genomic structural variants that lead to specific and mutually exclusive oncogenic activation of *GFI1B* and *GFI1* by repositioning these next to super-enhancers (a process called ‘enhancer-hijacking’) [93]. These structural variants are restricted to Group 3 and Group 4 tumors [93]. Growth factor-independent 1b (*GFI1B*) and its paralog *GFI1* are transcriptional repressors that function as key regulators of hematopoiesis [94] and were suggested to recruit histone-modifying enzymes to promoters and enhancers of target genes. In an orthotopic xenograft model, Gfi1/Gfi1b cooperates with c-MYC to drive medulloblastoma, despite the fact that neither alone is sufficient to promote tumorigenesis [93]. The resulting tumors are highly proliferative and metastatic, and exhibit histological and molecular characteristics consistent with human Group 3 medulloblastoma. In human tumors, only GFI1 activation, but not GFI1B, significantly correlates with MYC expression, despite the fact that both genes cooperate with MYC in the in vivo model [93]. Although a smaller set of tumors from the GTML model shows a resemblance to Group 4 medulloblastoma and MYCN-driven postnatal stem cells give rise to tumor cells that are positive for the Group 4 KCNA1 marker [20], there are as yet no reliable Group 4 models for medulloblastoma. However, most recently Lin et al. described the nature of the regulatory landscape in a set of human medulloblastoma samples [95]. Using this screen, they could identify master regulators like LMX1A, EOMES, and LHX2, which where differentially regulated active enhancers in Group 4 medulloblastoma [95]. It is suggested that these genes could be used in the search for finding the cellular origin of Group 4 medulloblastoma, which would be useful when generating novel Group 4 models.

To our knowledge there are no mouse models that recapitulate the other MYC-driven childhood brain tumors, e.g., MYC-driven DIPGs. Still, with the use of PDX models, brain tumor entities not described in this section can be further studied and maintained in vivo. PDX models are particularly useful in modeling slow-growing or otherwise hard-to-culture brain tumor cells and are very useful when evaluating novel drugs in vivo.

## 6. MYC Target Genes Involved in Tumor Maintenance and Recurrence

MYC-driven tumors are maintained by MYC expression and often show dependence on a continued activity of MYC proteins, a process commonly referred to as oncogene addiction. Such an addiction is neatly illustrated in chronic myeloid leukemia (CML) patients carrying *BCR-ABL* chimeric genes caused by translocations that can be effectively inhibited with BCR-ABL tyrosine kinase inhibitors like imatinib [96]. The process of oncogene addiction could be observed in the large set of inducible MYC and MYCN-models generated [6].

In normal embryonic stem cells, a pluripotent dormant state is induced upon MYC depletion [97]. Similarly, only highly quiescent, dormant hematopoietic stem cells survive the depletion of both c-*MYC* and *MYCN* genes, while committed hematopoietic progenitors are lost due to impaired proliferation, differentiation, and apoptosis [98]. By contrast, neural stem cells in c-*MYC/MYCN* double-knockouts are decreased in number, showing suppressed cell cycling capability and migration [99]. Dormant tumor cells are also found when depleting MYC in some tumor types, including epithelial mammary and hepatocellular tumors [100,101]. Here these dormant tumor cells later give rise to tumors that are independent of MYC or have become refractory to MYC inhibition. By contrast, in some models of lymphoma [71,102] and papillomatosis [103], MYC depletion instead gives rise to cell cycle arrest, apoptosis, and complete regression. In osteogenic carcinomas [104], MYC depletion gave rise to differentiation into normal bone. Interestingly, upon MYC reactivation in this model, such differentiated bone cells were rapidly forced into apoptotic cell death [104]. Similarly, MYCN-driven GTML brain tumors display a rapid onset of senescence and regression of tumor proliferation upon MYCN depletion [88]. In cured animals only smaller remnants of differentiated tumor cells could be found, even after several months of MYCN reactivation.

This variation in response to oncogenic depletion is not straightforward and therefore hard to explain. One possible mechanism for the variation is that some tumor models are clonal but others are polyclonal. This suggests that during the massive overexpression of *MYC* genes over a long period, multiple clones arise with various sets of additional genetic and epigenetic mutations. During this selective process it is possible that MYC-independent clusters might arise. Another mechanism could be related to the cellular context and the fact that certain cells of tumor origin have a certain set of collaborating genetic and epigenetic alterations that either promote or inhibit tumor cell death upon *MYC* gene depletion. As previously mentioned, MYC can partner up with RAS in driving tumorigenesis [55]. The variation can also be explained by the fact that MYC amplifies the output of activated gene expression programs in a cell, which thus depends on the set of genes that are active in a particular cell type or a certain stage in development [105,106]. Despite these differences, there are, as previously discussed, a few particularly important genes that control cell cycle checkpoints, apoptosis, and/or senescence, including *p53*, *p14^ARF^*, and *BCL-2* [56,107,108], which can interfere with MYC depletion in various MYC-driven tumor models.

Apart from tumor cell-intrinsic effects, alterations in the immune surveillance can affect tumor maintenance and the response to *MYC* depletion. Host-dependent mechanisms can cause lymphoma regression from *MYC* inactivation but only in immunocompetent hosts [80]. Suppression of *MYC* via Omomyc further induced changes in the microenvironment of pancreatic tumor cells [109]. Interestingly, from a perspective of oncogene addiction, MYC can make non-MYC-driven tumors dependent on MYC signaling as Omomyc can inhibit RAS-driven lung tumors [110,111].

*MYC* genes often emerge as late events in tumor progression and at tumor recurrence. Moreover, *MYC* gene amplification or increased MYC activity is often correlated with aggressive tumor phenotypes and poor outcomes in many cancers [112]. Just to give a few examples, *MYC* alterations and amplifications are poor prognostic markers or late events in our three most common types of cancer, lung cancer [113], prostate cancer [114], and breast cancer [115]. *MYC* gene mis-expression also often arises after failed therapies and then presumably promotes tumor recurrence. For example, *MYCN* amplifications in combination with *AURKA* amplifications commonly (~40% of cases) arise in relapsed neuroendocrine prostate cancer after hormonal therapy [116]. In addition, *c-MYC* is a radiosensitive locus that is altered by translocations or amplifications following radiation therapy of breast cancer cells, emphasizing its role in radiogenic breast cancer progression and recurrence [117]. Although the examples above are mostly in adult cancers, a recent report showed by studying matched primary and recurrent patient samples that *c-MYC* and *MYCN* amplifications in combination with p53 defects could also frequently emerge during medulloblastoma recurrence in children [89].

## 7. Pharmacological Inhibition of MYC Proteins and Their Transcriptional Targets

Transcription factors (TFs), such as the MYC family, are notorious for their involvement in several key cellular processes and extensive undruggability. Due to the pleiotropic nature and obscure biophysical properties of TFs, direct-target drug development has been stagnant over the past decades. Generally speaking, it is difficult to target intracellular proteins that lack enzymatic activity, which is why targeting kinases and cell surface proteins has long been the primary goal of therapy development.

**Figure 2:**
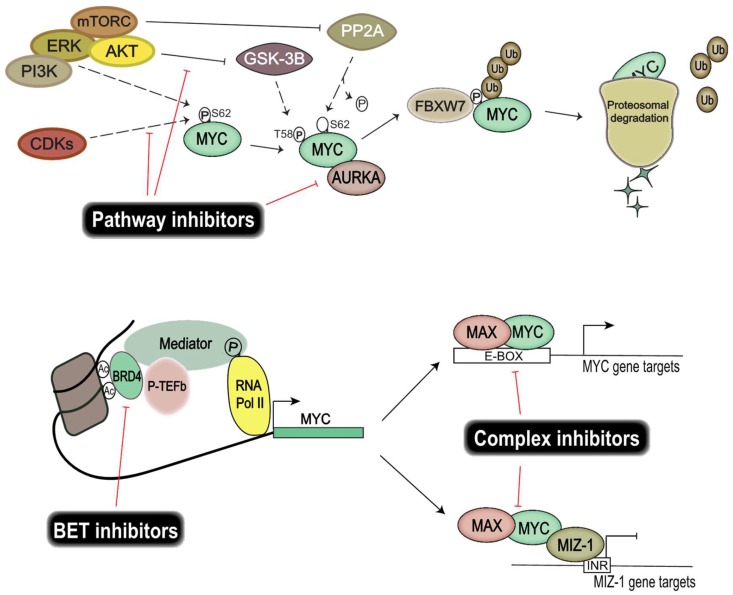
Schematic representation of ways of targeting MYC and its transcriptional output using specific signaling pathway, BET bromodomain, and complex inhibitors in MYC-dependent malignancies. Pathway inhibitors (top) target key proteins important for MYC-protein stability and activity. BET bromodomain inhibitors (lower left) compete with BRD4 in binding acetylated histone residues and thus halt the initiation of *MYC* transcription and subsequent regulation of MYC target genes. Complex inhibitors (lower right) disrupt complex formation and interaction with complex binding sites. MYC/MAX heterodimers bind to enhancer regions (E-BOX elements) and stimulate gene activation. Interaction of MYC/MAX together with MIZ-1, binding to the initiator (INR) region, promotes gene repression [146]. Disruption of these interactions would diminish gene target regulation and hence tumor progression in MYC-dependent malignancies.

The recent focus on genomic and epigenomic drug targets has opened up important alternative ways of targeting TFs, without the need for direct protein interference (Figure 2). Instead, efforts are directed at disrupting the transcriptional regulatory units essential for transcription of TFs. Epigenetic bromodomain inhibition emerged as an effective way to target oncogenic drivers such as MYC, by disrupting BET (bromodomain and extra-terminal) bromodomain interaction with polyacetylated histone tails [118]. JQ1 and iBET were the first line of inhibitors that proved efficacious in targeting MYC and its transcriptional output [118,119] by disrupting RNA polymerase II activity at the enhancer and superenhancer regions of *MYC* genes. However, these compounds resulted in toxicity in vivo and thus are not suitable for clinical use. Fortunately, modified BET bromodomain inhibitors such as TEN-010 and OTX105 are currently evaluated in clinical trials (NCT01987362, NCT02259114). Moreover, targeting histone deacetylase (HDAC), responsible for histone hypoacetylation and gene silencing, has proven fruitful in models of Group 3 medulloblastoma [120].

Interestingly, targeting heterodimeric MYC complexes has once again become an area of intense focus. MYC and MAX heterodimers are essential for transcriptional activation and oncogenic transformation of cells. A study from Wang et al. showed that celastrol triterpenoid derivatives selectively bind to MYC dimers and prevent them binding to DNA [121]. These derivatives inhibit the proliferation of a number of human cancer cell lines, including those resistant to bromodomain inhibition, and show low in vivo toxicity. Moreover, MYC–MIZ-1 complexes have been shown to repress genes important for neuronal differentiation in MYC-dependent Group 3 medulloblastoma, thus allowing tumor cells to retain a stem-like state, so targeting this complex would also be of therapeutic interest [92].

MYC protein stability facilitates uncontrolled induction of cellular proliferation and growth of cancer cells. Finding and exploiting targets important for MYC protein stability would allow us to control MYC activity. Several key players are investigated for their use as therapeutic targets, among them CDKs, Aurora kinase A, and PI3K/Akt. CDK and cyclin complexes are good candidates for therapeutic targeting due to their regulatory role in the cell cycle. Similarly to many chemotherapy drugs, CDK inhibitors halt cell proliferation by disrupting progression in the cell cycle. Moreover, CDKs also play an important part in regulating the protein stability of MYC proteins. CDK1 inhibition induces MYC-dependent apoptosis in various tumor cells [122] and the overexpression of *MYC* activates CDK2 and increases cyclin A/E gene expression [123,124]. Similarly, CDK2 suppresses the cellular senescence that is induced by c-MYC [125]. These interactions exemplify the intricate relationship between MYC and CDKs, and demonstrate a potential benefit from disrupting these feedback loops therapeutically, since CDK/cyclin complexes are also found frequently deregulated in cancers [126]. Palbociclib, a CDK4/CDK6 inhibitor, is currently evaluated in clinical trials for different solid tumors and has been approved for the treatment of breast cancer [127]. Several studies have shown that Palbociclib targeted neoplastic cells in breast cancer [128,129] and glioma [130,131], and that treatment resulted in an increased sensitivity to radiotherapy in medulloblastoma [132]. Another CDK inhibitor, Milciclib, which targets CDK2, has shown great promise in a phase I clinical trial on solid tumors [133]; whether this inhibits MYC function is, however, not yet known. Molenaar et al. demonstrated that the inactivation of CDK2 leads to synthetic lethality in MYCN-overexpressing neuroblastomas [134], thereby re-establishing CDK2 as a viable therapeutic target in MYC-dependent malignancies.

Other targets of interest are Aurora kinases, which are serine/threonine-protein kinases known to be involved in essential processes of mitosis. It has been shown that MYC upregulates expression of Aurora kinases A and B in neoplasms [135], and that Aurora kinase A is known to stabilize MYCN protein in neuroblastoma [136]. Interestingly, here Aurora kinase A interacts with MYCN but also with FBXW7, which ubiquitinylates MYCN, leading to suppressed degradation of the protein. Aurora kinase A inhibition has been shown to further increase the chemosensitivity of medulloblastoma cells [137], leading to a potential for reducing the dose of damaging chemotherapy. Ahmad et al. demonstrated that inhibition of Aurora kinase A, using MLN8237, converts MYCN-addicted GTML neurospheres to resemble non-MYCN expressors and that in vivo treatment significantly prolongs the survival of allografted mice [138]. In a *Trp53*-deficient liver cancer model, Dauch et al. showed that MYC directly binds to Aurora kinase A, and inhibition of their interaction by MLN8237 results in MYC degradation and cell death [139]. Highlights of the available literature suggest that targeting Aurora kinase A is an effective way of disrupting MYC stability in c-MYC/MYCN-dependent malignancies including medulloblastoma.

Excessive PI3K pathway signaling in cancer cells has displayed a modulation of Akt downstream target GSK-3β activity. MYC activity is dependent on phosphorylation of the S62 residue, which is negatively regulated by GSK-3β and mTOR. Aberrant PI3K signaling thus results in an increased half-life of MYC proteins, suggesting that restoration of GSK-3β activity using inhibitors directed toward PI3K signaling will disrupt tumor growth. The PI3K/mTOR inhibitor NVP-BEZ235 selectively killed MYCN-expressing neuroblastoma tumor cells through apoptosis and concomitantly eliminated MYCN protein in vivo [140]. OSU03012 is another PI3K/Akt inhibitor that leads to activation of GSK-3β. Targeting the PI3K/Akt pathway using OSU03012 has proven advantageous in both neuroblastomas [141] and medulloblastomas [142], showing a reduced MYC transcriptional output as well as affecting the stability of MYC proteins.

An evolving strategy to target MYC is to stabilize G-quadruplex DNA structures using small molecules. G-quadruplexes are four-stranded nucleic acid structures that may form in guanine-rich areas and can adopt various topologies. They can be stabilized in specific topologies that disrupt certain biological processes [143]. G-quadruplex sequences have been found in promoter regions of, for example, *MYC*. Today, scaffolds based on an indolylmethyleneindanone pharmacophore, which specifically stabilizes the parallel topology of promoter quadruplex DNAs, have demonstrated a specific disruption of *c-MYC* and *c-Kit* promoter regions [143]. Further studies on these small molecules are however needed to properly evaluate their applicability in biological systems.

Reiterating what several studies have concluded, due to the strong oncogenic potential and elusive nature of MYC proteins, combination therapy or dual targeting is probably the best way to target MYC-driven tumors. Sun et al. recently showed that a dual HDAC and PI3K inhibitor, CUDC-907, downregulated MYC and suppressed the growth of MYC-dependent neoplasms [144]. Another study by Pei et al. showed a synergistic killing of MYC-dependent medulloblastoma cells when combining HDAC and PI3K inhibitors [145], an effect partly ascribed to the induction of the tumor suppressor FOXO1. As a final point, despite the many ways to effectively target MYC-driven tumors by indirect MYC drug targeting (Figure 2), the exact mechanism behind this targeting is not always evident.

## 8. Conclusions

In this review, we highlighted the mechanisms behind MYC-driven tumor initiation, maintenance and recurrence with a focus on malignant childhood brain tumors. Despite numerous articles on the subject, it is still not clear how MYC and its regulatory network are promoting such entirely different processes in cancer. By summarizing the data reported from quite a few sophisticated MYC-driven brain tumor models, it is increasingly evident how context-dependent these various cancer processes are.

Dysregulated MYC proteins cannot alone drive and promote cancer. Instead, these transcription factors are part of and coordinate a network of various sets of substrates or putative partners that are active during MYC-driven initiation, maintenance, and recurrence. It is also clear that better targeting of MYC-dependent tumors with more direct MYC-targeting compounds or novel combinations of drugs suppressing MYC proteins synergistically would help to achieve better therapeutic responses. However, although not discussed in this review, better MYC drugs would still meet an unavoidable obstacle in brain tumor therapy—the blood–brain barrier (BBB) [147]. Interestingly, the BBB is disrupted in certain types of WNT-driven medulloblastomas, a subgroup that generally expresses high levels of MYC [148]. In these brain tumors, BBB disruption is maintained by WNT inhibition. It is therefore tempting to suggest combining WNT antagonists to open up the BBB with MYC therapy in order to more effectively deplete MYC-dependent brain tumors and block their recurrence.
